# α-Synuclein propagation leads to synaptic abnormalities in the cortex through microglial synapse phagocytosis

**DOI:** 10.1186/s13041-023-01059-1

**Published:** 2023-10-17

**Authors:** Dayana Pérez-Acuña, Soo Jean Shin, Ka Hyun Rhee, Sang Jeong Kim, Seung-Jae Lee

**Affiliations:** 1 Department of Biomedical Sciences, Seoul National University College of Medicine, 103 Daehak-Ro, Jongro-Gu, Seoul, 03080 Republic of Korea; 2 Department of Physiology, Seoul National University, College of Medicine, Seoul, 03080 Republic of Korea; 3 Neuroscience Research Institute, Seoul National University College of Medicine, Seoul, 03080 Republic of Korea; 4 Convergence Research Center for Dementia, Seoul National University College of Medicine, Seoul, 03080 Republic of Korea; 5 Neuramedy, Seoul, 04796 Republic of Korea; 6 Present Address: Department of Biochemistry, Molecular Biology and Biophysics, University of Minnesota, Minneapolis, MN 55455 USA

**Keywords:** Parkinson’s disease, α-synuclein, Protein aggregation, Microglia, Synapse degeneration

## Abstract

The major neuropathologic feature of Parkinson’s disease is the presence of widespread intracellular inclusions of α-synuclein known as Lewy bodies. Evidence suggests that these misfolded protein inclusions spread through the brain with disease progression. Changes in synaptic function precede neurodegeneration, and this extracellular α-synuclein can affect synaptic transmission. However, whether and how the spreading of α-synuclein aggregates modulates synaptic function before neuronal loss remains unknown. In the present study, we investigated the effect of intrastriatal injection of α-synuclein preformed fibrils (PFFs) on synaptic activity in the somatosensory cortex using a combination of whole-cell patch-clamp electrophysiology, histology, and Golgi-Cox staining. Intrastriatal PFF injection was followed by formation of phosphorylated α-synuclein inclusions in layer 5 of the somatosensory cortex, leading to a decrease in synapse density, dendritic spines, and spontaneous excitatory post-synaptic currents, without apparent neuronal loss. Additionally, three-dimensional reconstruction of microglia using confocal imaging showed an increase in the engulfment of synapses. Collectively, our data indicate that propagation of α-synuclein through neural networks causes abnormalities in synaptic structure and dynamics prior to neuronal loss.

**Supplementary Information:**

The online version contains supplementary material available at 10.1186/s13041-023-01059-1.

## Introduction

α-Synuclein is a 14-kDa protein enriched in the presynaptic terminal, where it associates with other synaptic proteins and participates in SNARE-complex assembly [1], synaptic vesicle pool trafficking, and neurotransmitter release [2]. Most research related to α-synuclein is related to its involvement in Parkinson’s disease (PD) and other neurodegenerative diseases, reflecting the fact that this protein is the major constituent of intraneuronal inclusions known as Lewy bodies, a hallmark of such disease. A large body of evidence has firmly established that α-synuclein aggregates are key contributors to the pathogenesis of PD and other synucleinopathies. These aggregates form in a few discrete regions of the brain and spread widely to other regions as the disease progresses [3]. However, the consequences of aggregate spreading on neural function remains unknown.

There has been a recent upsurge in research on α-synuclein–dependent early synaptic alterations based on evidence that these alterations precede neuronal degeneration. The impact of α-synuclein aggregation on neuronal function has been studied extensively in mouse and cell models overexpressing wild-type α-synuclein [4, 5] or mutated forms of α-synuclein known to cause early-onset familial PD [6, 7]. These reports have demonstrated neurotransmitter release defects, mitochondrial dysfunction, abnormal calcium homeostasis, dopamine depletion, and impaired long-term potentiation (LTP) [2]. However, these studies did not consider the involvement of extracellular forms of α-synuclein, which would affect neural function during the process of interneuronal propagation.

Preformed fibrils (PFFs) are now extensively used to model aggregate spreading and investigate the pathological effects of propagated α-synuclein. Reports have shown that exposure to oligomeric and α-synuclein PFFs elicits defects in neuronal function, such as defects in neuronal excitability and connectivity [8], impaired long-term potentiation [9], and dendritic spine pathology [10, 11]. However, the causal relationships among these abnormalities are not well understood.

In this work, we describe structural and functional synaptic abnormalities in the somatosensory cortex—a region to which α-synuclein aggregates spread to in the PFF mouse model. We also show that increased microglial phagocytosis is a potential causal mechanism underlying synapse loss in this region.

## Results

### Intrastriatal injection of α-synuclein fibrils causes cortical accumulation of phosphorylated α-synuclein and gliosis

**Fig. 1:**
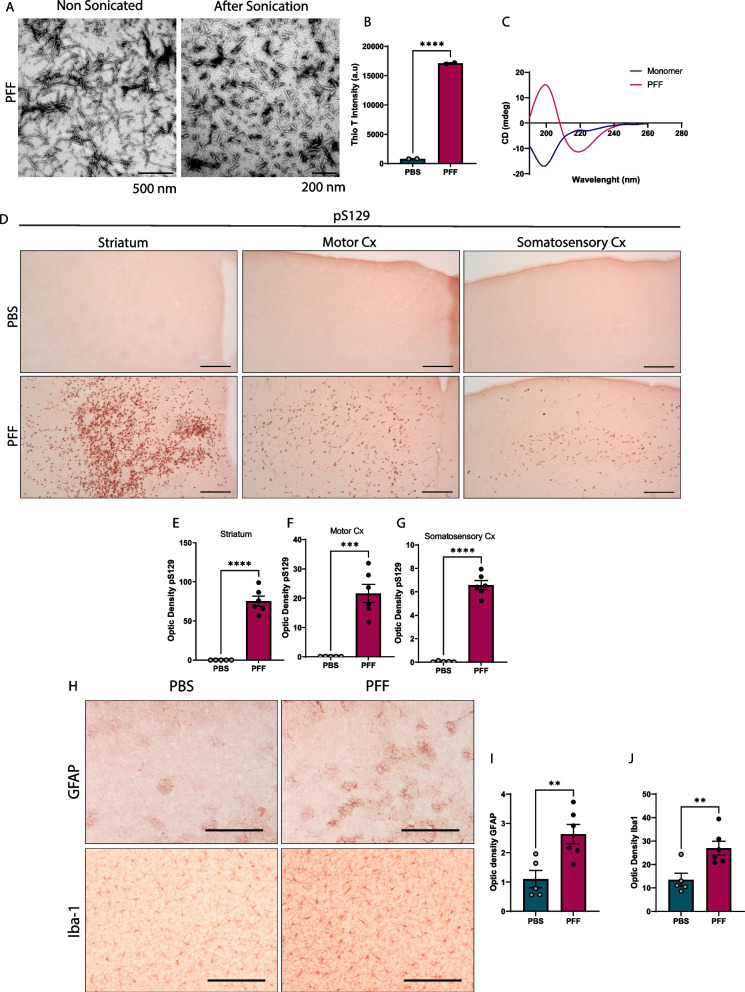
Characterization of PFFs and brain pathology induced by PFF injection. **A** TEM images of mouse PFFs before and after sonication. Scale bars = 500 nm (before) and 200 nm (after). **B** Thioflavin-T intensity of fibrils 7 days after incubation (n = 2 measurements). **C** CD spectra of α-synuclein monomers and PFFs. **D** Representative images of brain immunohistochemical staining for pS129-α-syn in striatum, motor, and somatosensory cortices 20 weeks post injection (p.i.). Scale bar = 100 µm. **E**–**G** Quantification of pS129-α-syn optical density in striatum (**E**), motor (**F**), and somatosensory (**G**) cortex. **H** Glial activation in the somatosensory cortex. Representative images of brain astrocytes and microglia 20 weeks after injection, identified by GFAP and Iba1 immunostaining, respectively. **I**, **J** Quantification of GFAP (**I**) and Iba1 (**J**) immunoreactivity in the somatosensory cortex. Data in **B**, **E**–**G**, **I** and **J** are expressed as means ± S.E.M. (**B**, n = 2 for PBS and PFF; **E**–**G**, **I** and **J**, n = 5 and 6 mice for PBS and PFF, respectively; *p < 0.05, **p < 0.01, ***p < 0.001; Student’s t-test)

To investigate the effects of α-synuclein propagation in synaptic transmission, we used the PFF mouse model. Characterization of assembled mouse PFFs using thioflavin T binding assay, circular dichroism (CD), and transmission electron microscopy (TEM) showed that these fibrils had properties similar to those described elsewhere [12], with enriched β-sheet fibrillar structure and protein fragments of ~ 50–100 nm after sonication (Fig. 1A–C).

To validate our propagation model, we examined the accumulation of phosphorylated α-synuclein (pS129) in several mouse brain regions after intrastriatal injection. Similar to previous reports [3, 13], immunohistochemical staining showed an increase in immunoreactivity against pS129-α-syn, mostly in the striatum (injection site) and cortical areas, 20 weeks after injection (Fig. 1D). In addition to the injection site and track, inclusions were detected across the somatosensory cortex, mostly in layer 5 and below layer 1. We focused on this brain area instead of striatal or motor cortex regions to distinguish spreading-related neuronal changes from alterations caused directly by PFFs.

Given that aggregation of pS129-α-syn is often accompanied by activation of astrocytes and microglia and contributes to neurodegeneration [14], we assessed the expression of glial fibrillary acid protein (GFAP) and ionized calcium-binding adapter molecule 1 (Iba1) in the somatosensory cortex (Fig. 1H) and found an increase in the expression of both markers (Fig. 1I, J).

### Accumulation of pS129-α-syn leads to cortical synapse loss

**Fig. 2:**
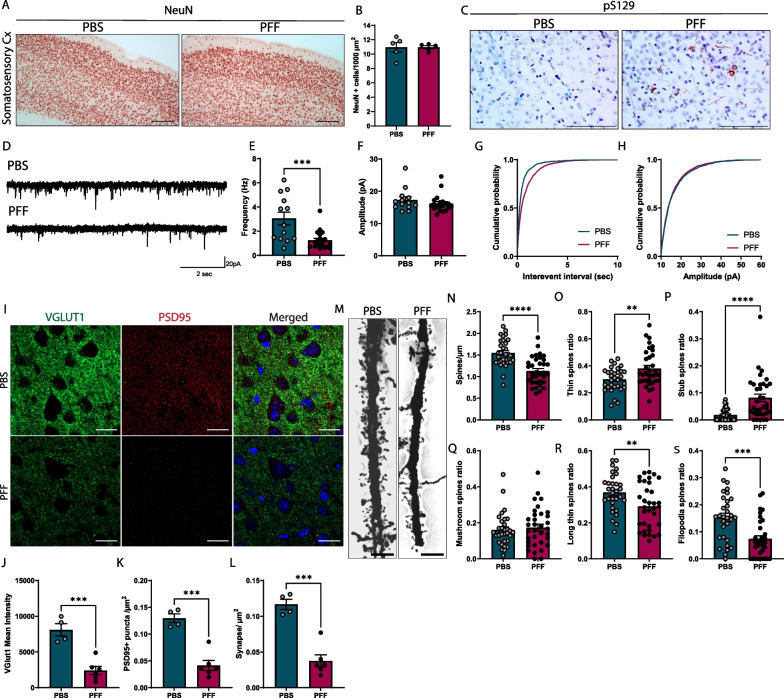
Synaptic pathology in the somatosensory cortex layer 5 following PFF injection. **A** Representative images of NeuN-stained neurons in the somatosensory cortex 20 weeks post injection (p.i.). Scale bar = 100 µm. **B** Quantification of neuronal density in the somatosensory cortex. **C** Localization of pS129-α-syn aggregates in layer 5. Scale bar = 100 µm. **D** Traces showing sEPSC recordings from neurons in the same layer. **E** Frequency of sEPSC events (PBS = 3.06 ± 0.53 Hz; PFF = 1.26 ± 0.17 Hz). **F** Amplitudes of sEPSC events (PBS = 17.29 ± 0.99 pA; PFF = 16.19 ± 0.61 pA). **G** Cumulative probability of sEPSC interevent interval. **H** Cumulative probability of sEPSC amplitude. **I** Images of synapses in layer 5, identified by immunodetection of the presynaptic marker VGLUT1 and postsynaptic marker PSD95. Scale bar = 20 µm. **J** Quantification of VGLUT1 intensity. **K** Quantification of PSD95 puncta/µm^2^. **L** Quantification of synapse density (synapses/µm^2^) by co-localization of VGLUT1 and PSD95. **M** Golgi staining of apical dendritic spines from layer 5 pyramidal neurons. Scale bar = 5 µm. **N** number of spines per micron. **O**–**S** Ratio of spines: thin (**O**), stub (**P**), mushroom (**Q**), long thin (**R**), and filopodia (**S**). Data are expressed as means ± S.E.M. (**A**–**C**, n = 5 and 4 mice for PBS and PFF, respectively; **D**–**H**, n = 13 and 20 neurons for PBS and PFF, respectively, from 4–5 mice; **I**–**L**, n = 4 and 6 mice for PBS and PFF, respectively; **M**–**S** (PBS, n = 31 and 34 neurons for PBS and PFF, respectively, from 5 mice; *p < 0.05, **p < 0.01, ***p < 0.001; Student’s t-test)

To assess the functional consequences of aggregate spreading, we examined synaptic function in the somatosensory cortex following PFF injection. First, to corroborate that synaptic impairment was not the product of neuronal loss, we addressed the state of neuronal degeneration 20 weeks after injection. An examination of the somatosensory cortex showed no differences in the number of neurons positive for neuronal nuclear protein (NeuN), a marker of post-mitotic neurons (Fig. 2A, B). To further investigate whether propagation of α-synuclein rather than PFF itself has an impact on synaptic activity, we measured spontaneous excitatory postsynaptic currents (sEPSC) in layer 5 of the somatosensory cortex—the layer containing pS129-α-syn inclusions (Fig. 2C). We observed a decrease in the frequency but not amplitude of sEPSCs recorded from pyramidal neurons at 20 weeks post injection (Fig. 2D–H). We also measured evoked excitatory postsynaptic currents (eEPSCs) and paired pulse ratio (PPR) in layer 5 of the somatosensory cortex. eEPSCs amplitude was measured after stimulation from 10 to 120 µA from layer 5 to layer 5. Despite the decrease in synapse density, eEPSCs amplitudes were similar between PFF-injected mice and controls (Additional file 1: Fig. S1B, C). Similarly, PPRs showed no differences between experimental groups at inter-stimulus intervals of 30–150 ms (Additional file 1: Fig. S1D, E).

To better understand the mechanistic basis for synaptic dysfunction, we first considered the possibility that the decrease in spontaneous events was attributable to a decrease in synapse density. Quantification of synapses using the presynaptic marker, Vglut1 (vesicular glutamate transporter 1), and postsynaptic marker, PSD95 (post-synaptic density protein 95), confirmed the loss of excitatory synapses in layer 5 of the cortex of PFF-injected mice (Fig. 2I–L). Similarly, [15] using Golgi-Cox staining, we found spine pathology in the same layer, specifically a decrease in spine number and changes in spine morphology (Fig. 2M). For the most part, we found a decrease in long thin (> 1 µm length) spines and filopodia (> 2 µm length), which are known to be more transient and unstable, after PFF injection, and an increase in thin and stub spines (Fig. 2N–S). Interestingly, we found no differences in mushroom spines—more stable and persistent spines that are involved in long-term potentiation [16]. This would suggest that α-synuclein pathology primarily affects newly formed and dynamic synapses.

To determine whether synaptic alterations resulted in behavioral changes, we assessed cognitive and motor functions. We found that spatial working memory was impaired in the Y maze, with no signs of motor dysfunction or exploratory behavior in rotarod and open-field tests, respectively (Additional file 1: Fig. S2A–D).

**Fig. 3:**
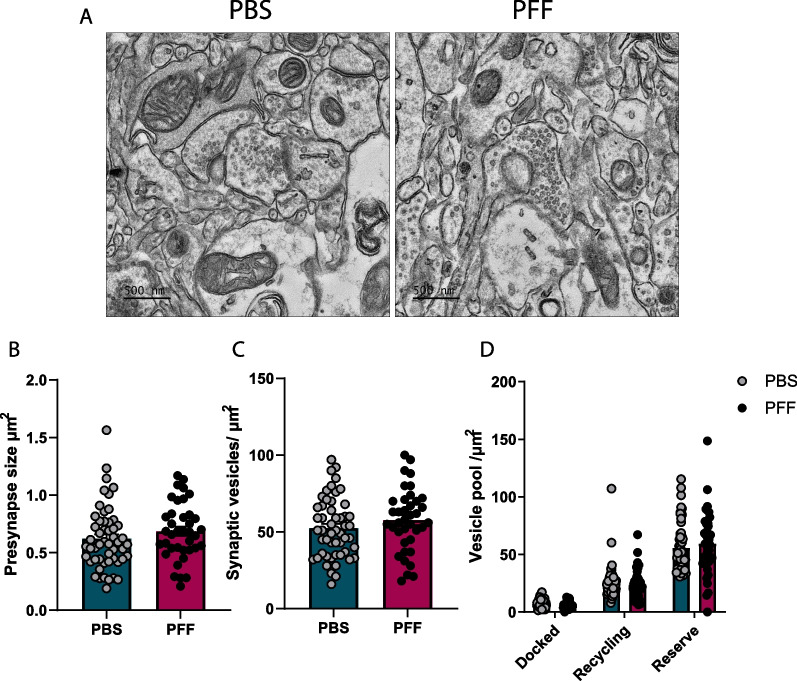
No change in synaptic vesicle number or vesicle pool. **A** Representative EM images of synapses from PBS and PFF mice. Scale bar = 500 nm. **B** Size of presynaptic terminals (µm^2^). **C** Synaptic vesicle numbers (vesicles/µm^2^). **D**. Quantification of synaptic vesicles docked and in recycling and reserve pools (vesicles/µm^2^). Data are expressed as means ± S.E.M (**B**–**D**, n = 52 and 38 synapses for PBS and PFF, respectively, from 5 mice; *p < 0.05, **p < 0.01, ***p < 0.001; Student’s t-test)

Additionally, we characterized synaptic vesicle homeostasis in the somatosensory cortex using electron microscopy (EM). A subsequent quantitative analysis showed no changes in the total number of synaptic vesicles in the presynaptic compartment. An analysis performed after classification of synaptic vesicles into three pools – releasable, recycling, and reserve – also showed no differences in the presynaptic compartment. These results confirm that accumulation of α-synuclein in the cortex does not affect neurotransmitter release (Fig. 3A–D).

### Phagocytosis of synapses is increased in the cortex of PFF-injected mice

**Fig. 4:**
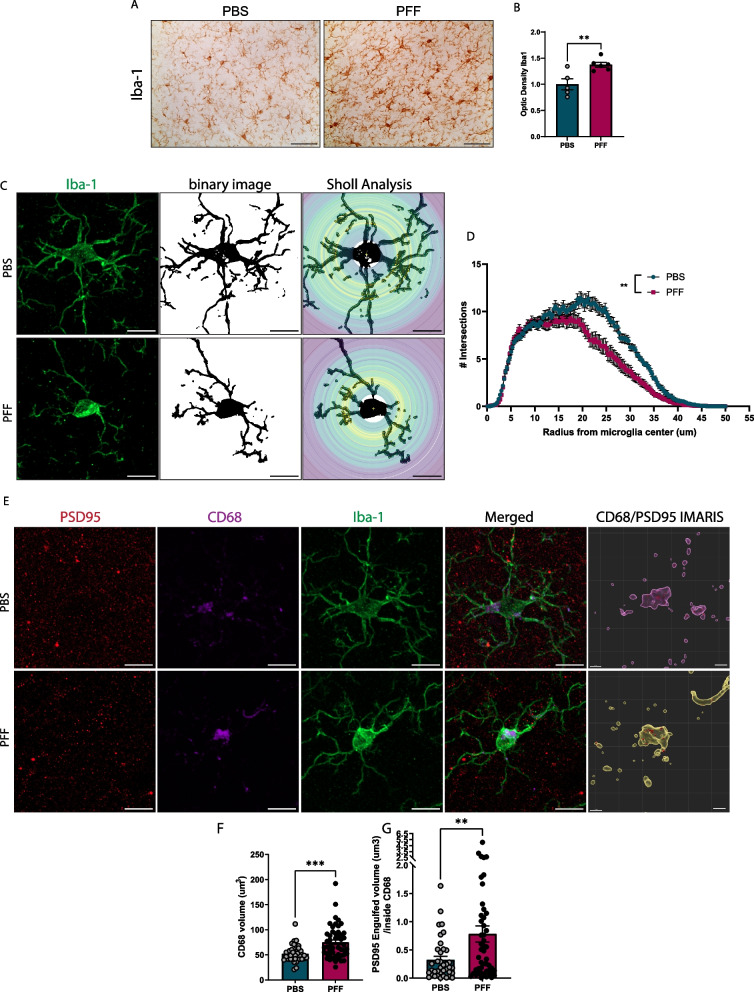
Increased microglial synapse phagocytosis. **A** Increased microgliosis in somatosensory cortex layer 5, detected as Iba1 expression. **B** Quantification of optical density of Iba1 staining in layer 5. **C** Representative images of individual microglia for Sholl analysis. Scale bar = 10 µm. **D** Number of intersections per radius from microglia centers. **E** 3D reconstruction of PSD95 engulfed inside lysosomal CD68 compartments; scale bar = 10 µm. CD68/PSD95 IMARIS; scale bar = 2 µm. **F** Quantification of CD68 microglial volume. **G** Quantification of PSD95 engulfed volume inside CD68. Data in **B**, **D**, **F**, and **G** are expressed as means ± S.E.M. (n = 37 and 49 microglia for PBS and PFF, respectively, from 5 mice; *p < 0.05, **p < 0.01, ***p < 0.001; Student’s t-test)

Microglia mediate synapse remodeling and regulate synaptic density under physiological conditions and during neurodevelopment [17]. Dysregulation of microglia phagocytosis, including disruptions in the balance between “eat me” and “do not eat me” signals, in neurodegenerative diseases have established a link between protein aggregation and the synaptopathy caused by increased synapse engulfment [18–20]. A Sholl analysis of microglia in layer 5 of the cortex of PFF-injected mice revealed retracted processes compared with PBS-injected mice, measured as a reduction in the number of intersections (Fig. 4C, D), which is known to be associated with inflammatory phenotypes, secretion of cytokines, and phagocytic activity. These observations are consistent with the known relationship between microglial morphology and the function and activation state of microglia [21]. To further investigate the role of microglia in cortical synapse alterations after PFF injection, we performed a three-dimensional (3D) confocal reconstruction of microglia and quantified the content of postsynaptic marker, PSD95, within CD68-positive compartments (Fig. 4E). First, we found an increase in the volume of CD68-positive compartments within Iba-1–stained microglia, indicating increased clearance activity (Fig. 4F). We then examined PSD95 within CD68-positive lysosomal compartments in cortical layer 5 of PFF-injected animals and found that PSD95 content increased in these compartments (Fig. 4G). Because complement activation and localization of C3 and C1q complement factors in synapses has been implicated in aberrant microglia mediated synapse loss in Alzheimer’s disease (AD) models [19, 22], we asked whether complement factor C1q was upregulated in the cortex of PFF-injected mice. However, we found no evidence of increased expression or co-localization of C1q with postsynaptic markers in layer 5 (Additional file 1: Fig. S3), indicating that the synapses that are eliminated are not tagged by the classical complement cascade. Collectively, these findings suggest that the synapse loss is mediated by microglial phagocytosis of cortical synapses independent of complement activation during α-synuclein spreading.

## Discussion

Altered sensory motor function, reflecting tactile and proprioceptive changes, are often seen in PD patients during early stages of the disease [23]. However, the effects of pathological α-synuclein on network function in the cortex have not been extensively addressed. Here, we demonstrate that injection of mouse α-synuclein PFFs into the striatum induced α-synuclein deposition, synapse loss and impairment, and dendrite pathology in layer 5 of the somatosensory cortex. These changes in the cortex were accompanied by increased microglial phagocytosis of synapses, consistent with the idea that this phagocytic process is the cause of synapse loss. The results of the current study thus suggest that α-synuclein propagation leads to synapse loss and dendrite remodeling, at least in part, through increased microglial synapse phagocytosis. These synaptic changes are emblematic of disturbances in network function caused by the pathological spreading of α-synuclein during the course of disease.

The effects of PFFs on cortical synaptic transmission have previously been addressed in vitro by treating primary cultures or brain slices with α-synuclein fibrils. These studies have revealed that exogenous α-synuclein induces Lewy body pathology and lead to a decrease in synaptic protein, reduced synaptic activity, and altered spine dynamics prior to neuron loss [11, 12, 24]. Some studies have shown that α-synuclein PFFs and oligomers directly interact with membranes and synaptic vesicles, modulating calcium transients and vesicle homeostasis [25–27]. Here, we assessed the synaptic changes that occur during the propagation of α-synuclein in a neural network by measuring sEPSCs in the somatosensory cortex after PFF injection into the striatum, finding a decrease in the frequency of spontaneous events and a concomitant decrease in synapse density. These results are consistent with previous findings obtained in hippocampal primary neurons [24]. Interestingly, although the number of synapses was reduced in cortices of PFF-injected mice, we found no changes in eEPSCs, suggesting that additional mechanisms might act to compensate for this reduction and maintain evoked synaptic strength. The decrease in synapse number could be compensated by functional changes at the remaining synapses, such as through increases in neurotransmitter release or upregulation of postsynaptic receptors. Decreases in postsynaptic density accompanied by enhanced presynaptic function owing to a larger number of docked vesicles in synaptic terminals has been observed after PFF treatment *in* *vitro* [11]; however, we found no difference in either synaptic vesicle pools or PPR 20 weeks after PFF injection. Given that α-synuclein oligomers are known to trigger glutamate release from astrocytes and activate extra-synaptic NMDA receptors [28], the release of glutamate from a source other than neurons is another mechanism that could increase excitatory responses and maintain synaptic strength. Alternatively, decreases in inhibitory inputs, as has been observed in vivo after chronic exposure to PFFs, causes hyperactivity in layer 2/3 neurons of the somatosensory cortex [29]. Overall, these studies support the idea that pathological α-synuclein could affect synaptic function through multiple mechanisms producing varying outcomes.

We further observed dendritic spine abnormalities in the cortex, suggesting that α-synuclein propagation causes detrimental changes in spine turnover and architecture. In agreement with these findings, two-photon imaging studies reported by Blumenstock et al. revealed lower spine density in cortical layers 4/5 and 1 after intrastriatal PFF injection [15].

Microglia, the main neuroimmune cells in the brain, promote neuronal well-being and function by sensing the environment, protecting neurons from injurious agents, and performing physiological housekeeping functions. Synapse remodeling, which is necessary for CNS development and circuit homeostasis, is among these latter functions in which microglia are involved [30]. Dysregulation of microglial homeostasis leading to increased phagocytic activity has been linked to engulfment of neurons and synapses and subsequent cognitive deficits in mouse models of AD [20, 31, 32] and multiple sclerosis [33]. In the present study, we showed that the propagation of pathological α-synuclein after intrastriatal PFF injection causes excessive removal of synapses in the cortex. Microglial engulfment may not be the sole cause of synapse degeneration during α-synuclein propagation. Other studies have suggested that PFF also impacts the formation and molecular organization of presynaptic terminals by inhibiting β-neurexin [34] or by decreasing the levels of soluble α-synuclein [35]. However, it is worth noting that the latter studies examined the direct effects of PFFs on neurons in culture, whereas our study assessed the effects of α-synuclein propagation throughout a neural network in an intact-tissue environment. In addition, we also observed astrogliosis in layer 5. It has been shown that astrocytes participate in synapse removal during development, adult brain and disease [18, 36] and when exposed to oligomeric α-synuclein induce synapse loss by releasing glutamate and enhancing extrasynaptic NMDAR activity [28]. Therefore, further studies should be carried out to clarify the contribution of astrocytes in synapse degeneration in the PFF model.

In conclusion, our current research demonstrated that the propagation of α-synuclein through a neural network causes deficits in synaptic structure without neuronal loss, and that these synaptic changes might be mediated by microglial-mediated synapse engulfment. Future research on how synapses are tagged for microglial removal would pave the way to a deeper understanding of the functional and structural defects caused by α-synuclein propagation through neural networks.

## Materials and methods

### Generation of recombinant α-synuclein and preparation of mouse PFFs

Mouse wild-type α-synuclein monomers were purified as described previously [37] and subsequently lyophilized and stored at − 80 °C. Lyophilized α-synuclein was reconstituted in Dulbecco’s phosphate-buffered saline (DPBS; a1285601 Gibco, Carlsbad, CA, USA), filtered through a 100-kDa membrane (Nanosep; OD100C34; Pall Life Sciences, Port Washington, NY, USA). PFFs were prepared by adjusting the concentration of purified monomers to 5 mg/ml and incubating them at 37 °C for 7 days with constant shaking at 1000 rpm. PFFs were stored at − 80 °C until the day of injection, at which time the fibrils were thawed at room temperature and sonicated for a total of 30 s (60 pulses, 1 s on, 1 s off) at 20% power (Vibracell VCX130; Sonics, Newtown, CT, USA).

### Transmission electron microscopy

For visualization of fibrils and characterization of the structure of the injected material, 5 mg/ml of PFFs was diluted 1:50 in PBS, after which 10 µl was adsorbed onto 200-mesh carbon-coated copper grids (Electron Microscopy Sciences, Hatfield, PA, USA), air dried for 5 min, and negative-stained by incubating with 10 µl of 2% uranyl acetate (Cat. Number U1006; Spectrum Chemical, Brunswick, NJ, USA) for 5 min. Fibrils were observed using a JEM-1400 transmission electron microscope (JEOUL; Akishima, Tokyo, Japan).

### Circular dichroism spectroscopy

Circular dichroism (CD) spectra of protein samples (monomers and fibrils) between 190 and 260 nm were obtained in 0.1-mm cells with a step resolution of 1.0 nm, bandwidth of 1.0 nm, and scan speed of 100 nm/min using a Chirascan Plus spectropolarimeter (Applied Photophysics, Leatherhead, Surrey, UK). All obtained spectra were averages of 10 separate measurements.

### Thioflavin T (Thio T) binding assay

Forty microliters of a diluted PFF sample were added to 50 μl of 10 μM Thio T (#T3516; Sigma Aldrich, St. Louis, MO, USA) solution in glycine (#BP3815; Fisher Scientific, Hampton, NH, USA)–NaOH (pH 8.5). After a 5-min incubation, fluorescence was measured at excitation and emission wavelengths of 450 and 490 nm, respectively.

### Animals and stereotaxic injection of PFFs

All animal experiments were performed on male wild-type C57BL/6N mice in accordance with the standards of the Seoul National University Institutional Animal Care and Use Committee (IACUC, SNU-190417-2-6). For intrastriatal injection of PFFs, 8-week-old male C57BL/6N mice were anesthetized intraperitoneally with ketamine/xylazine and a total volume of 2.5 µl of PBS or PFFs (2 mg/ml) was injected stereotaxically into the right striatum (AP, 1.0 mm; ML: 1.5 mm; DV, 3.0 mm) at a rate of 0.5 µl/min using a 30G needle.

### Sample collection

Brain samples were collected after anesthetizing mice by intraperitoneal administration of 200 mg/kg of 1.25% Avertin (2,2,2 tribromoethanol, T48402; Sigma Aldrich) and then perfusing them with a 0.9% NaCl (saline) solution. For samples used for histological analysis, mice were also perfused with 4% paraformaldehyde (PFA) following saline solution.

### Brain immunohistochemistry

Free-floating brain sections (40-µm-thick) were washed with PBS and incubated with 3% H_2_O_2_ for 1 h. After peroxidase quenching, samples were washed and blocked with PBS containing 4% bovine serum albumin (BSA) in 0.1% Triton X-100 and incubated overnight at 4 °C with primary antibodies against pS129-α-syn (ab51253, 1:500; Abcam, Waltham, MA, USA), Iba-1 (019–19741, 1:1000; Wako Fujifilm, Richmond, VA, USA), GFAP (ab7260, 1:1000; Abcam), and NeuN (MAB377, Clone A60, 1:1000; Chemicon, Merk Millipore, Burlington, MA, USA). The next day, sections were washed three times with PBS containing 0.1% Triton X-100 (PBST) and then incubated first with biotinylated secondary antibodies (1:2000; Vector Laboratories,Newark, CA, USA) and then with avidin–biotin-peroxidase complex (Vectastain ABC kit, PK6200; Vector Laboratories), followed by staining with DAB (D5637; Sigma Aldrich). Brain sections were then mounted on gelatin-coated slides, and brain regions, identified using the Allen Mouse Brain Atlas and imaged using a Zeiss AX10 brightfield microscope (Carl Zeiss, Germany). The total area corresponding to the brain region of interest in the coronal slice was used for analysis. Immunoreactivity (intensity) and NeuN-positive neuron densities were analyzed using Image J open-source software (National Institutes of Health [NIH], MD, USA). For intensity measurements, the same threshold was applied to all images.

### Immunofluorescence and synapse quantification

Free-floating brain sections (40-µm-thick) were washed with PBS and blocked by incubating with PBS containing 10% goat serum, 2% BSA, and 0.5% Triton X-100 for 4 h at room temperature. Slices were then incubated with primary antibodies against PSD95 (ab18258 or ab12093, 1:500: Abcam); Vglut1 (135304; Synaptic Systems, Goettingen, Germany), or pS129-α-syn (clone 81A; Millipore) in blocking solution for 48 h at 4 °C. Thereafter, brain slices were incubated with Alexa 488-conjugated anti-guinea pig, Alexa 647-conjugated anti-rabbit, or Cy3-conjugated anti-mouse (1:1000) secondary antibodies, as appropriate, for 4 h at room temperature, then washed with PBST, incubated with 4′,6-diamidino-2-phenylindole (DAPI; 1:5000) for 10 min, and slide-mounted with Prolong Gold Antifade (p36930; Invitrogen, Waltham, MA, USA). Images were acquired with a Zeiss LS900 Confocal microscope equipped with a Plan-Apochromat 63x/1.40 Oil DIC M27 objective. Synapses were quantified using ImageJ software (Fiji edition; NIH) using the method described by Rebollo et al. [38].

### Microglia reconstruction for Sholl and synapse phagocytosis analyses

Free-floating brain sections (40-µm-thick) were washed with PBS and blocked by incubating with PBST containing 10% goat serum, 2% BSA and 0.5% Triton X-100 for 4 h at room temperature. Slices were then incubated with primary antibodies against PSD95 (ab12093, 1:250; Abcam), Vglut1 (135304; Synaptic Systems), Iba-1 (019-19741, 1:1000; Wako), or CD68 (MCA1957, 1:500; Bio-Rad, Hercules, CA, USA) in blocking solution for 48 h at 4 °C. Thereafter, brain slices were incubated with Alexa 488-conjugated anti-guinea pig, Alexa 448-conjugated anti-rabbit or Alexa 647-conjugated anti-goat secondary antibodies, as appropriate, or stained with rhodamine Red X (1:1000), for 4 h at room temperature, then washed with PBST, incubated with DAPI (1:5000) for 10 min, and slide-mounted with Prolong Gold Antifade (p36930; Invitrogen). Engulfed content was analyzed using IMARIS software; image processing and quantification of engulfed volume was performed as described previously [39]. For Sholl analyses, microglia intersections were quantified using a Sholl Analysis plugin, as described elsewhere [40].

### Golgi-Cox staining

Golgi staining was performed as described previously [41, 42] with slight modifications. Briefly, mouse brains were collected after saline perfusion, separated into two hemispheres, and immersed in Golgi-Cox solution for 24 h at room temperature in the dark. The next day, the solution was replaced with a fresh solution and stored in the dark at room temperature for 13 days. Brains were then rinsed with distilled water and transferred to tissue protectant solution overnight at 4 °C, after which the solution was again replaced with fresh solution and incubated for 6 days at 4 °C. Ipsilateral hemispheres were sectioned into 150-μm coronal sections using a vibratome. Sections were mounted onto gelatin-coated slides and dried for 4 days. Staining was performed by washing tissue sections twice with distilled water for 5 min each and then incubating in ethanol for 5 min, after which tissue was transferred to a 3:1 ammonia solution and incubated for 8 min. Samples were then washed twice with distilled water and incubated in 5% sodium thiosulfate for 10 min in the dark, followed by dehydrating with a 70%, 95%, 100% ethanol series and xylol (5 min each). Finally, sections were mounted using Eukkit mounting media.

Z-stack images of segments of apical dendrites from pyramidal neurons in layer 5 of the somatosensory cortex located at 100 μm of neuron soma were acquired with a Leica DM5500B microscope (Leica, Wetzlar, Germany) equipped with a 100 × oil-immersion objective and running on Las X ver 3.7 software. Quantitative analyses of the number of dendritic spines in a 30-µm segment were performed using open source Reconstruct software; six apical dendrites from 5 mice per group were analyzed. Spine density was quantified and parameters were classified as previously described [43]

### Electrophysiology

For electrophysiological recordings, performed 20 weeks after injection, mice were anesthetized with isoflurane and decapitated, and their brains were rapidly removed and sliced into 300-µm slices containing the somatosensory cortex using a vibratome (VT1200s; Leica). Slices were cut in ice-cold cutting solution containing 93 mM *N*-methyl-d-glucamine (NMDG), 2.5 mM KCl, 10 mM MgSO_4_, 0.5 mM CaCl_2_, 1.25 mM NaH_2_PO_4_, 30 mM NaHCO_3_, 25 mM glucose, 20 mM HEPES, 5 mM Na ascorbate, 2 mM thiourea, 3 mM Na pyruvate and 12 mM l-acetyl-cysteine, perfused with 95% O_2_ and 5% CO_2_ (300 mOsm, pH 7.34). Slices were transferred immediately to cutting solution at 32 °C, incubated for 10 min, and then transferred to artificial cerebrospinal fluid (ACSF) consisting of 125 mM NaCl, 2.5 mM KCl, 1 mM MgCl_2_, 2 mM CaCl_2_, 1.25 mM NaH_2_PO_4_, 26 mM NaHCO_3_ and 10 mM glucose, perfused with 95% O_2_ and 5% CO_2_. Recordings were performed after incubating in ACSF for 1 h at room temperature.

Whole-cell recordings were acquired from neurons in layer 5 of the somatosensory cortex. Pyramidal cells were identified using differential interference contrast optics. Whole-cell patch-clamp recordings were performed using glass pipettes (4–5 MΩ) filled with an internal solution with the following composition: 135 mM K-gluconate, 5 mM KCl, 2 mM NaCl, 10 mM HEPES, 0.1 mM EGTA, 5 mM Mg ATP, 0.4 mM Na, 3 mM GTP and 10 mM Tris (di) phosphocreatine (pH 7.20, adjusted with KOH). For recordings, brain slices were placed in a submerged chamber and perfused with ACSF at 32 °C. After obtaining the whole-cell configuration, neurons were clamped at − 70 mV for 5 min. sEPSC were recorded at a holding potential of − 70 mV. Series resistance was monitored at the beginning and end of the experiment. Cells with a resistance series greater than 25 MΩ were excluded.

Data were acquired using an EPC-10 patch amplifier (HEKA Electronik) and PatchMaster software (HEKA Electronik), sampled at a rate of 20 kHz and filtered at 2 kHz. For sEPSCs, 150 events from each neuron were detected and analyzed using Minhee Analysis [44]. eEPSCs and PPRs were elicited in the presence of 50 µM picrotoxin using a stimulating electrode filled with ACSF placed in layer 5 approximately 100 µm from the recorded neuron. Average eEPSCs were calculated from five consecutive traces. PPRs were elicited at 50% of the maximal response and calculated from the mean amplitude of the EPSC evoked by the second stimulus divided by the mean amplitude of the eEPSC evoked by the first stimulus.

### Synapse electron microscopy

Brain samples were collected, and tissue was fixed in 2% PFA/1% glutaraldehyde in cacodylate buffer. Following fixation, the tissue slices were treated with 1% OsO4 and 1.5% potassium ferrocyanide in 0.1 M phosphate buffer (pH 7.3) for 1 h at 4 °C in the dark before being embedded in Epon 812 after dehydration in ethanol and propylene oxide serial soaking. Two days were spent polymerizing using pure Epon 812 resin at 70 °C. Ultrathin sections of 60 nm thickness were produced with an ultramicrotome (EM UC7, Leica, Austria) and collected onto 100-mesh copper grids. Subsequently, the sections were stained with 2% uranyl acetate (8 min) and lead citrate (5 min) and observed at 120 kV using the KBSI Bio-HVEM System (JEM-1400 Plus; JEOL, Tokyo, Japan). All analyses were performed by measuring the area of presynaptic boutons with a visible postsynaptic density. For pool classification, synapses were analyzed as previously described [45]. Briefly, compartments were delineated at 80-nm intervals. Docked vesicles were counted as those in contact with the active zone (postsynaptic density), and vesicles withing 160 nm of the active zone were considered to belong to the recycling pool. Reserve pool vehicles were classified as those outside the 160-nm–delimited compartment. Vesicles were counted using the Cell Counter plugin on Image J.

### Statistical analysis

Results in figures represent means ± standard error of the mean (S.E.M.). Statistical significance was determined by calculating p-values using unpaired Student’s t-tests or analysis of variance (ANOVA), as appropriate. Statistical analyses were performed, and graphs were drawn using GraphPad Prism 9.2.0 (GraphPad Software Inc., La Jolla, CA, USA).

### Supplementary Information

**Additional file 1: Figure S1**. eEPSC and PPR in layer 5 neurons show no alterations. **Figure S2**. Behavioral alterations 20 weeks after PFF injection. **Figure S3**. C1q levels in layer 5 of the somatosensory cortex.

## Data Availability

The datasets used and/or analyzed during the current study are available from the corresponding author on reasonable request.

## Abbreviations

PD: Parkinson’s disease
PFF: Preformed fibrils
pS129-α-syn: α-Synuclein phosphorylated at serine 129
TEM: Transmission electron microscopy
EM: Electron microscopy
CD: Circular dichroism
IBA1: Ionized calcium-binding adapter molecule 1
GFAP: Glial fibrillary acidic protein
CD68: Cluster of differentiation 68
NeuN: Neuronal nuclei
ACSF: Artificial cerebrospinal fluid
PSD95: Post synaptic density 95
VGLUT1: Vesicular glutamate transporter 1
sEPSC: Spontaneous excitatory postsynaptic current
eEPSC: Evoked excitatory postsynaptic current
PPR: Paired-pulse ratio
NMDA: *N*-Methyl-d-aspartic acid
AMPA: α-Amino-3-hydroxy-5-methyl-4-isoxazelopropionic acid
C1q: Complement component 1q
