# Supplementary material for: α-Synuclein propagation leads to synaptic abnormalities in the cortex through microglial synapse phagocytosis
Source: Mol Brain. 2023 Oct 17;16:72. doi: 10.1186/s13041-023-01059-1 (PMC10580656; doi:10.1186/s13041-023-01059-1)
Supplement: Supplementary file 1 — Additional file 1: Figure S1. eEPSC and PPR in layer 5 neurons show no alterations. Figure S2. Behavioral alterations 20 weeks after PFF injection. Figure S3. C1q levels in layer 5 of the somatosensory cortex. [file 13041_2023_1059_MOESM1_ESM.pdf]

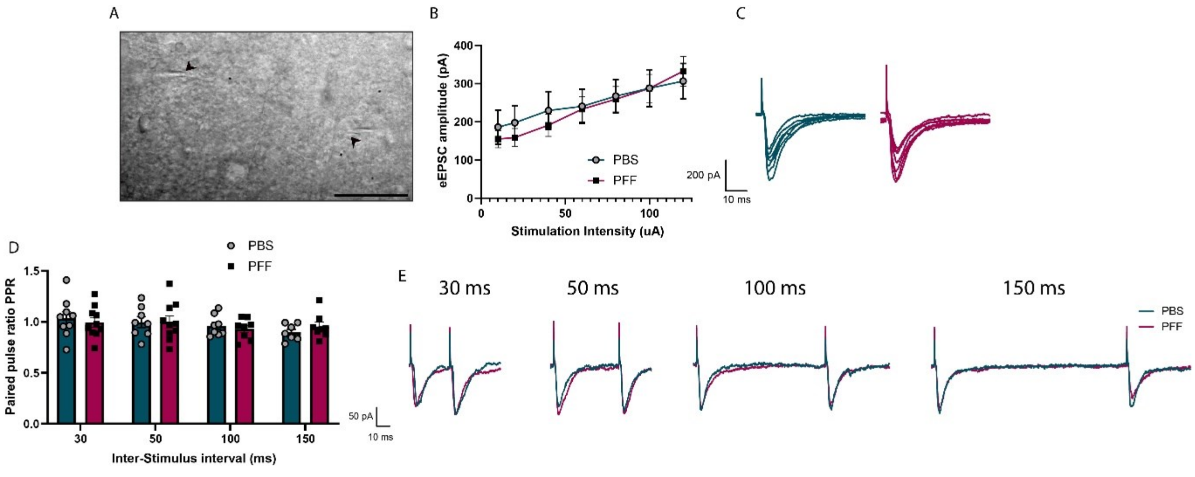

Figure S1. eEPSC and PPR in layer 5 neurons show no alterations.

A) Representative image of stimulation (left) and recording electrode (right). Scale bar 50 µm. B) Evoked EPSC amplitudes from PBS and PFFs injected mice. 10, 20, 40, 60, 80, 100, 120 µA (PBS= 186.22 ± 44.25, 197.67 ± 45.06, 229.44 ± 49.86, 240.56 ± 44.84, 267.03 ± 43.82, 287.94 ± 48.51, 306.59 ± 46.45 pA) (PFF =154.91 ± 22.70, 158.91 ± 23.60, 191.32 ± 29.34, 233.15 ± 33.24, 259.25 ± 33.44, 287.40 ± 36.87, 332.59 ± 38.82 pA) C) Traces of eEPSC D) Paired pulse rations from PBS and PFF mice. 30 ms, 50 ms, 100 ms, 150 ms.(PBS = 1.04 ± 0.06, 0.99 ± 0.05, 0.96 ± 0.04, 0.90 ± 0.03 ratio)(PFF= 0.99 ± 0.05, 1.00 ± 0.06, 0.93 ± 0.04, 0.96 ± 0.04) E) Traces of paired pulses after stimulation in layer 5. Data in B, D are expressed as means ± S.E.M. B-C (PBS, n=8-9; PFF n=10-12 neurons from 3 mice). Data in D (PBS, n=5; PFF n=5). *$p < 0.05$, **$p < 0.01$, ***$p < 0.001$; (B,D) Student's t-test.

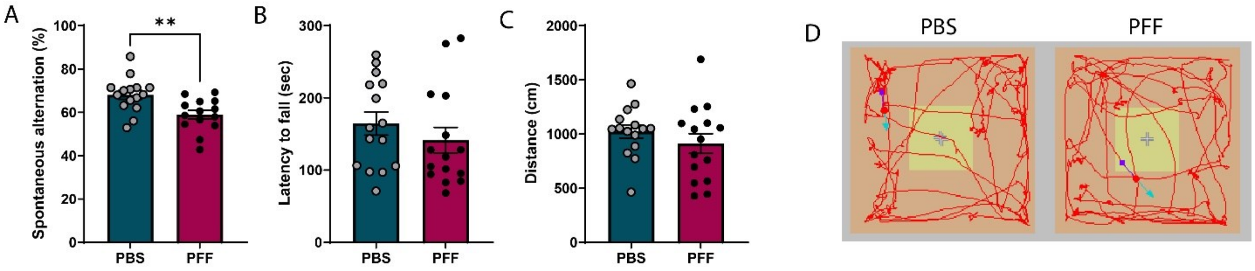

Figure S2. Behavioral alterations 20 weeks after PFF injection. A) Y maze spontaneous alteration percentage B) Latency to fall in the rotarod test. C) Open field distance (cm). D) Diagram of open field movement trace. Data in A-C are expressed as means ± S.E.M. A-C (PBS, n=15; PFF n=14 mice). *p < 0.05, **p < 0.01, ***p < 0.001; Student's t-test

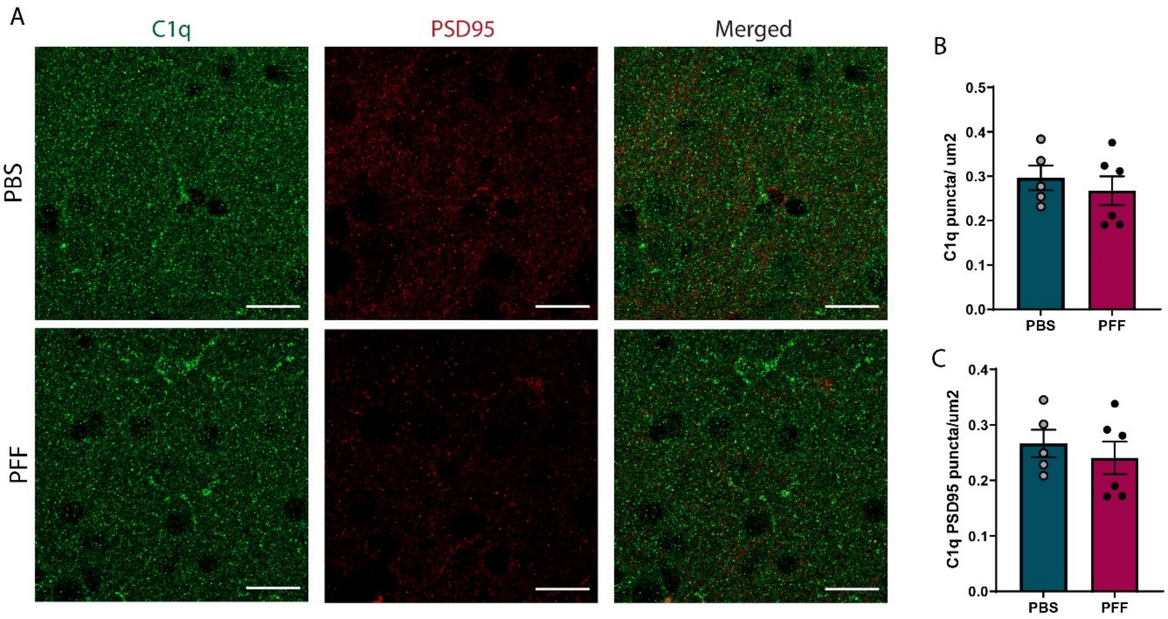

Figure S3. C1q levels in layer 5 of the somatosensory cortex. A) Representative immunofluorescence images against C1q (green) and PSD95 (red) B) Quantification of C1q puncta in layer 5. C) Quantification of C1q co-localization with postsynaptic terminals. Data in B-C are expressed as means ± S.E.M. (PBS, n=5; PFF n=6 mice). $*p < 0.05$, $**p < 0.01$, $***p < 0.001$; Student's t-test
